# Detecting Unidentified Changes

**DOI:** 10.1371/journal.pone.0084490

**Published:** 2014-01-13

**Authors:** Piers D. L. Howe, Margaret E. Webb

**Affiliations:** School of Psychological Sciences, University of Melbourne, Parkville, Victoria, Australia; University of Sussex, United Kingdom

## Abstract

Does becoming aware of a change to a purely visual stimulus necessarily cause the observer to be able to identify or localise the change or can change detection occur in the absence of identification or localisation? Several theories of visual awareness stress that we are aware of more than just the few objects to which we attend. In particular, it is clear that to some extent we are also aware of the global properties of the scene, such as the mean luminance or the distribution of spatial frequencies. It follows that we may be able to detect a change to a visual scene by detecting a change to one or more of these global properties. However, detecting a change to global property may not supply us with enough information to accurately identify or localise which object in the scene has been changed. Thus, it may be possible to reliably detect the occurrence of changes without being able to identify or localise what has changed. Previous attempts to show that this can occur with natural images have produced mixed results. Here we use a novel analysis technique to provide additional evidence that changes can be detected in natural images without also being identified or localised. It is likely that this occurs by the observers monitoring the global properties of the scene.

## Introduction

When we view an everyday scene we often get the compelling impression that we are able to see all parts of the scene simultaneously and that we can form a detailed representation of it [1]. This illusion [2] is reinforced by the fact that, typically, any change to the scene is easily noticed because such changes are usually accompanied by visual transients that draw attention to the location of the change [1]. However, if these transients are masked by other, stronger, visual transients then large changes can be missed. For example, observers often miss large changes to a scene when these changes occur simultaneously with an eye blink, a saccadic eye movement, a “mudsplash”, an abrupt change in viewpoint or a temporary blanking of the screen [3]–[6]. This phenomenon, known as *change blindness*, is generally thought to at least partially reflect the limitations of visual attention [1], [5], [7]. For example, according to coherence theory, an observer forms a stable representation of only those portions of scene to which he attends [1]. If a change occurs to one of these areas, the change can be detected by comparing the changed region in the new image to the representation of the corresponding region of the original image. Conversely, a change to an unattended region of the image cannot be detected because there is no representation of that portion of the original image to serve as a point of comparison. A central prediction of coherence theory is that whenever a change is detected the observer will always know what has changed since the observer will necessarily have a representation of the corresponding portion of the original image.

Other studies have stressed the fact that humans have some knowledge of the statistics of the scene. For example, observers have been shown to be able to estimate the mean and distribution of a number features such as size [8], orientation [9] and direction of motion [10]. It has been suggested that knowledge of these statistics can be used to guide visual search [11], to rapidly categorize scenes [12] and, most relevantly, to detect changes to scenes [13]. While monitoring scene statistics may well reveal when a change has occurred, it is less obvious how an observer should use changes in scene statistics to determine which item or object in a scene has changed. This raises the interesting possibility that an observer may be able to detect when a change occurs without being able to either identify or localise the object in the scene that has changed.

A previous study has shown that change detection thresholds can be less than discrimination thresholds, at least for sinusoidal stimuli. Nakayama and Silverman [14] measured both the contrast required to detect the displacement of a vertical sinusoidal grating and the contrast required to discriminate the direction of its displacement. It was found that for small displacements the contrast sensitivity function for detection was approximately double that for discrimination, implying that if the contrast and magnitude of displacement of the grating was chosen judiciously then observers would be able to detect the displacement of a grating but be unlikely to discriminate its direction of displacement. While this is a clear example of a situation where an observer can reliably detect a change without being able to identify it, the evidence that a similar phenomenon can occur with natural stimuli has been more mixed.

Rensink [15] presented evidence that with natural images observers are sometimes able to detect that something has changed without being able to identify or localize what has changed. Observers viewed two alternating images separated by a blank interval. The observers pressed a key when they first detected a difference between the two images and then pressed the key again when they could see and identify what had changed. If the interval between the two key presses was reliably over one second the observer was labelled as *can-sense* whereas if it was less than one second the observer was labelled as *only-see*, indicating that these observers did not detect changes before seeing them. Thirty percent of observers reliably demonstrated *can-sense* behaviour. It was claimed that these observers were able to sense changes even when they could not identify them.

These findings were challenged by a subsequent study that showed that the number of observers categorized as *can-sense* depended on the exact temporal cut-off between a *can-sense* trial and a *only-see* trial [16]. Simons et al. [16] repeated the Rensink (2004) study but varied the minimum allowable time between the two button presses for a trial to be labelled as *can-sense*. Increasing this time from 1 second to 1.5 seconds caused over 80% of the *can-sense* observers to be reclassified as *only-see*. It was argued that the *can-sense* observers were merely the ones that used a verification strategy, pressing the response key for the first time when they first suspected that a change had occurred but waiting until they could verify that the suspected change was real before pressing the response key for a second time to report that they had actually seen the change. Since it took 1.28 seconds for a change to occur twice in the same direction in the original Rensink [15] study, any observer employing this verification strategy would necessarily take slightly more than one second between button presses. Consistent with this more mundane explanation it was found that the *can-sense* observers also tended to have more false positives than the *only-see* observers, sometimes reporting that they detected a change had occurred even in the catch trials were no change had in fact occurred. This finding is consistent with the notion that *can-sense* observers were merely those with a lower threshold for the initial response. Under this interpretation, detection and identification are mediated by the same process but correspond to different thresholds of evidence [16].

Other studies have provided evidence that detection and identification may be mediated by different processes. For example, a recent ERP study by Busch, Frund and Hermann [17] measured the EEG signals generated when observers viewed a change blindness stimulus. It was found that the N2pc component and a change-related positivity occurred only when observers were able to identify a change and was absent when observers were able to only detect that a change had occurred. Further evidence that detection and identification are mediated by different mental processes comes from a study of comparative visual search [18]. In comparative visual search, two images are shown simultaneously side by side and the observer is asked to identify any differences between the two images [19]. Galpin and colleagues found that on trials where observers were able to identify the difference, as opposed to just being able to detect that the two images were not identical, there was an increase in the number of comparative saccades and an increase in fixation duration.

In summary, there are good reasons to believe that observers should be able to detect changes by monitoring the statistics of the scene, but that monitoring the scene statistics may not provide enough information to identify which object in the scene was changed. However, the behavioural evidence that this actually happens in practice with natural scenes is mixed. Although observers do report sometimes being able to detect a change without being able to identify which object has changed [15], it is unclear whether this is due to a response bias [16]. The purpose of the current investigation is to provide a behavioural test of whether changes in a natural scene can be detected without being identified in a manner that avoids this potential response bias confound.

## Experiment 1: Evidence for change detection without identification

In this experiment we use a novel analysis technique to investigate whether observers are able detect changes without being able to identify them. Previous investigations utilized a flicker paradigm in which the observer was repeatedly shown the original image and the changed image [15], [16]. This raised the possibility that observers might have been employing a verification strategy; pressing the response button the first time when they thought that they might have seen a change and then waiting until they had seen the change a second time before pushing the response button a second time to indicate that they could confidently identify the change. In our paradigm we avoided this potential verification confound by using a one-shot design in which the original image and altered image were shown only once, so that the observers did not have the opportunity to verify their original hunches. In addition, we introduced catch trials where no change occurred so as to enable us to estimate each observer's personal guessing rate. Using the estimated guess rate we could then determine on how many trials we would expect that, by pure chance, the observer would be able to guess that a change had occurred and then not be able to identify what had changed. Finding more than this number of *only-sense* trials would constitute evidence that observers can reliably detect changes that they cannot identify, beyond what would be expected due to a guessing bias.

### Participants

Ten naïve observers participated in Experiment 1. Four were male and ages ranged from 19 to 43 years. All had normal or corrected to normal visual acuity and colour vision as verified by a Good-Lite® near vision eye chart and the 1984 concise edition of Ishihara's test for colour-blindness (Kanehara & Co Ltd, Tokyo, Japan).

### Ethics Statement

All work was conducted according to the principles expressed in the declaration of Helsinki. From all participants we obtained informed written consent. The University of Melbourne Psychological Sciences Departmental Health Ethics Advisory Group specifically approved this study and the manner for obtaining consent.

### Stimuli

**Figure 1:**
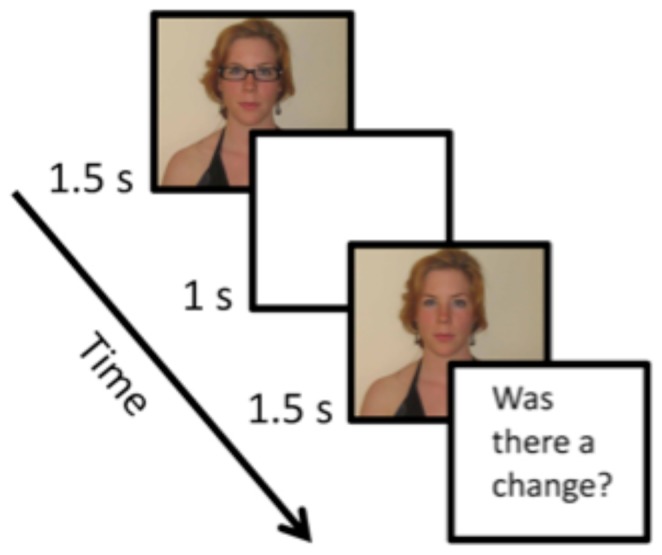
An example trial from the first experiment. Two portraits of the same individual were shown separated by a blank interval. The observer was first asked if a change occurred and, if they indicated that one had, they were then asked to indicate which change had occurred from a list of nine possible changes. The subject of the photograph has given written informed consent, as outlined in the PLOS consent form, to publication of their photograph.

The stimuli were presented via MATLAB 7.10 software (The MathWorks, Natick, MA) utilizing the Psychophysics Toolbox [20], [21]. In each trial, two portrait colour photographs were presented on a black background, both of the same individual, who was always female. The photographs were viewed on a CRT monitor with a resolution of 1280 by 1024 pixels, a frame rate of 85 Hz and displayed at a distance of 60 cm. Each image subtended approximately 20 by 27 degrees of visual angle. Each photograph was presented individually for 1.5 seconds with a 1 second blank interval separating the two photographs (Figure 1). After the second image was presented, the observer was first asked whether a change had occurred and had the option of clicking on the words “yes” or “no”. If the observer indicated that a change had occurred, he/she was then asked to identify the change by clicking on one member of a list of nine possible options (“Earrings”, “Necklace”, “Glasses”, “Hat”, “Lipstick”, “Eye Shadow”, “Eyeliner”, “Clothing”, and “Hair”).

### Creation of the photograph pairs

For each starting photograph the nine possible features were assigned random values of either zero or one using the MATLAB *rand* function. The subject of the photograph was then attired to match these random assignments, being asked to don or remove earrings, necklace, glasses etc as necessary. In the change trials, one of the nine possible options was selected at random and then changed. For example, if the hat option had been randomly selected to be changed and the subject of the photograph was not currently wearing a hat, then the subject would be asked to wear a hat for the second photograph. Thus, by looking at only one of the two photographs an observer had no way of reliably guessing whether or not a change had occurred. Changes could only be detected by comparing the two photographs.

In order to measure the observer response bias, it was necessary to include some catch trials where none of the nine possible changes occurred. These *no change* photographs pairs were constructed in a similar way as before. Each of the nine options were randomly assigned a number of either 0 or 1 and the subject of the photograph was then arranged to match these assignments. After the first photograph was taken, there was a short break during which the observer was asked move around, as though changing one of the available features, and then the second photograph was taken. This was done to ensure that the postures in the two photographs were not identical. It was necessary to do this because in a *change* trial the postures in the two photographs would not be identical either.

### Procedure

At the start of experiment, the observer was presented with written instructions explaining the task and shown an example trial for each of the nine possible changes. The observer was allowed to repeat each example trial as many times as required to clearly see the change. An example of a change would be the removal or addition of a pair of glasses, as shown below. As each change was different, they were not all equally difficult to identify.

In the main experiment, there were 100 trials where a change occurred and 40 catch trials where none of the nine possible changes occurred. Adapting the terminology of Rensink (2004) to our different experimental paradigm, for each observer we labelled a trial as *only-sense* if the observer correctly identified that a change had occurred but then misidentified which of the nine possible changes had occurred. Conversely, a trial was labelled as *can-see* if the observer was able to correctly identify which of the nine possible changes had occurred. By measuring the proportion of the catch trials where the observer incorrectly reported that a change had occurred, we could calculate for each observer how many *only-sense* trials could occur due to guessing, taking into account each observer's reporting bias. For each observer we subtracted this number from the total number of *only-sense* trials thereby ensuring that our findings could not be attributed to a reporting bias or a guessing strategy [22]. A derivation of the equation we used to compensate for observer bias is given below.

### Corrections for possible observer response bias

While the existence of *only-sense* trials would be evidence for the ability to detect unidentified changes, it is possible that an *only-sense* trial might occur due to guessing. Below, we derive an equation that predicts the number of *only-sense* trials that could occur due to a bias that the observer may have to respond ‘yes’ when asked if he/she noticed a change even when in reality he/she had not. We start by considering a hypothetical observer who has no ability to detect changes without being able to identify them. By definition, for this hypothetical observer, every time a change is detected the observer necessarily also knows what item has changed. For such an observer, we calculate the number of *only-sense* trials that would be expected to occur by chance due to a response bias to report that a change had been detected even when it had not.

We start by considering only those trials where a change has *actually occurred*. For this set of trials we define Y to be the number of these trials where the observer reported that a change had occurred (i.e., responded ‘yes’ (Y) at the end of the trial). We define S as the number of trials in which a change occurred and that the observer correctly detected, or ‘saw’ (S), the change. Similarly, we define G to be the number trials where a change occurred, the observer did not detect the change, but still reported that a change had occurred, i.e. guessed (G). Thus,
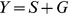
(1)


We further define A to be the number of trials where a change actually occurred and M to be the number of trials where a change occurred but was not detected by the observer, i.e. missed (M). It follows
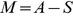
(2)


We define P to be the probability that an observer states that a change occurred even when he/she had not detected the change. Thus, P is the response bias. It follows that 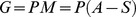
(3)


Substituting (1) into (3) and rearranging. We find 
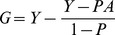
(4)


Having correctly guessed that a change has occurred, the observer is then presented with a list of possible changes and asked to indicate which of the possible changes actually did change. Let Q be the probability of incorrectly guessing which option had changed when the observer did not see the change, so has no idea what has actually changed. Since in Experiment 1 the observer is presented with a list of nine options it follows that 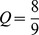
(5)


We define N to be the total number of *only-sense* trials, i.e. those trials where a change did occur, the observer responded that a change had occurred but then failed to correctly identify which change occurred. Because we have previously assumed that for our hypothetical observer if a change is seen then the observer will be able to correctly identify it (we consider alternative assumptions later), it follows that *only-sense* trials occur only when the observer did not see the change, so the equation for the number of *only-sense* trials is given by
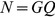
(6)


Substituting (4) into (6) we find
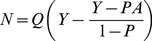
(7)


To estimate P, we consider those trials where a change did not occur. P is the proportion of those trials where the observer still reported that a change did occur, even though no change actually occurred. Using equation 7 we can then calculate the number of *only-sense* trials that would be expected to occur if our hypothetical observer has a response bias, i.e. tendency to report a change has occurred even when no change was detected. If the total number of *only-sense* trials exceeds the number predicted by equation 7 then this would be evidence that the observer can reliably detect changes that cannot be identified, even taking into account a possible response bias.

In equation (5), it was assumed that since the observer had not seen the change, the observer was guessing blindly at which change had occurred. However, it could be that the observer had some subconscious information as to what had changed, so his/her guesses would not be random [23]. This would mean that equation (5) would overestimate the number *only-sense* trials. As such, equation (7) represents an upper bound on the number of *only-sense* trials our hypothetical observer would be expected to make. Exceeding this upper bound therefore constitutes evidence that this observer can detect changes without being able to identify what has changed.

In estimating P we assumed that the probability of an observer responding that a change had occurred when a change was present but had not been detected is equal to the probability of an observer responding that a change had occurred when no change had occurred. We write this statement as
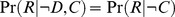
(8)where Pr stands for probability, R for responding that a change is present, D for the detection of a change and C for the presence of a change. For this statement to be true two assumptions must hold: (1) the observer's response depends on the detection of the change but not on the presence of the change itself and (2) a detection of a change cannot occur if there is no change because then there would be nothing to detect. If these two assumptions hold then equation (8) must hold. The proof is as follows. From the law of total conditional probability we can say
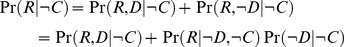
(9)


From assumption (1) it follows
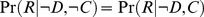
(10)


Combining (9) and (10) we find 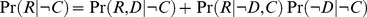
(11)


Pr(R|⌝C) equals Pr(R|⌝D,C) if Pr(R,D|⌝C) = 0 and Pr(⌝D|⌝C) = 1. If Pr(⌝D|⌝C) = 1 it follows that Pr(D|⌝C) = 0. Note that if Pr(D|⌝C) = 0 then it necessarily follows that Pr(R,D|⌝C) = 0 since Pr(R|D) is finite. In other words, we need to assume that if there is no change there can be no detection of the change. This does not mean that the observer may not still report that a change occurred, only that the observer did not actually detect the change. In the absence of the detection of a change, the observer may guess that a change occurred. Our analysis is designed to estimate this guessing rate.

### Results and discussion

**Figure 2:**
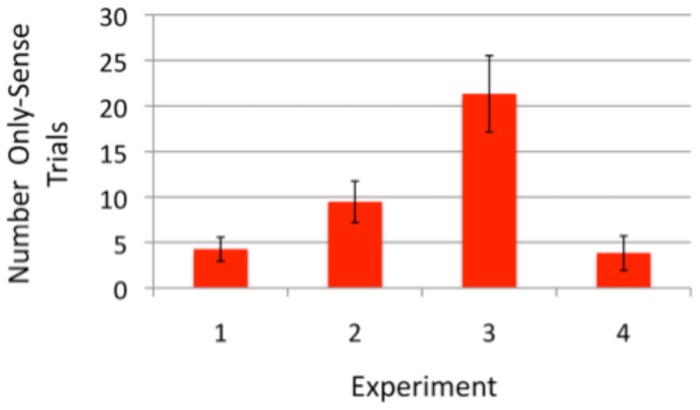
The number of *only-sense* trials, out of a total of 100 trials where a change occurred, corrected for possible observer response bias. Error bars represent the standard error of the mean.

**Figure 3:**
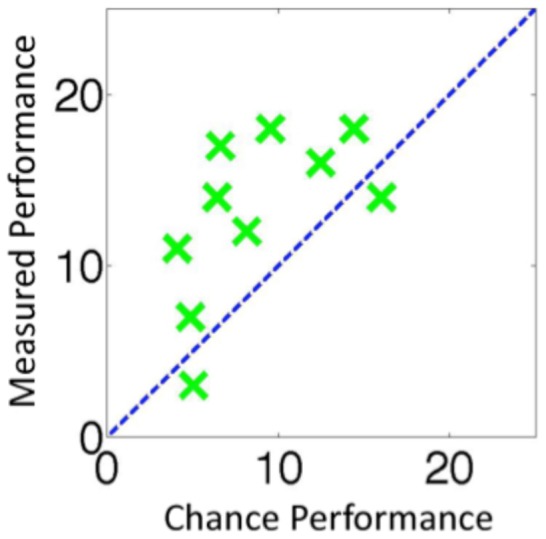
The results for individual observers for Experiment 1. The x-axis shows the maximum number of *only-sense* trials that could be attributed to observer bias (i.e. a guessing strategy) and the y-axis shows the number of *only-sense* trials that were actually measured. Data points above the dotted line constitute evidence that observers could detect changes that they could not identify.

Out of the 100 trials where a change occurred, observers were able to identify that a change had occurred on 73.0 of them. Of these trials, they were further able to identify correctly what had changed on 60.0 trials. This meant that on 13.0 of the trials where a change occurred, they were able to identify that a change had occurred but then were not able to correctly identify what had changed. From measuring the observers' guessing rate, we would have expected 8.7*only-sense*. As shown in Figure 2, the actual number of *only-sense* trials, in excess of those that could be attributed to an observer guessing strategy, is 4.3, which reliably exceeded zero *t(9)* = 3.04, *p* = 0.014, *Cohen's d* = 0.969. The data was arcsine transformed before performing the t-test to ensure normality [24]. The data for individual observers is shown in Figure 3. These results indicates that observers could sometimes detect changes without being able to identify them and that this finding could not be attributed to an observer response bias [16].

## Experiment 2: Inverting the face stimuli

It is thought that face stimuli are processed differently from other stimuli and that there is a particular brain area, the fusiform face area, responsible for their processing [25], [26]. As such, it is possible that the result from Experiment 1 may apply only to those stimuli that contain faces. It is known that this specialized, holisitic processing of faces can be reduced by inverting them [27]. We therefore repeated Experiment 1 with all the stimuli inverted so as to reduce the degree of holistic processing that change detection might rely on. As before there were ten observers, four male, ages ranging from 18 to 26 years old. All other aspects of the experiment were kept constant.

**Figure 4:**
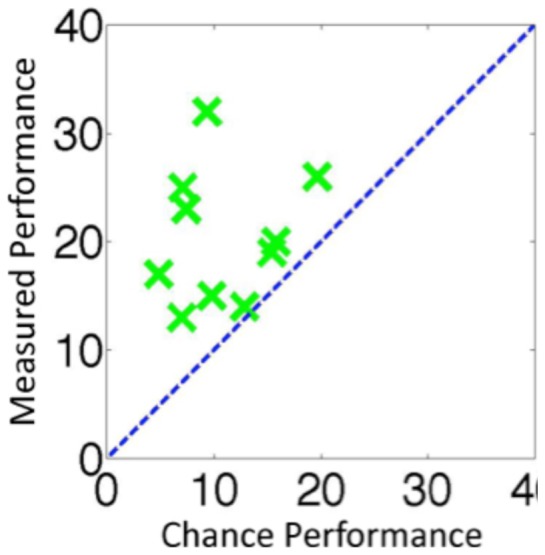
The results for individual observers for Experiment 2 using the same format as Figure 3.

The group results are shown in Figure 2 and the individual results in Figure 4. Out of the 100 trials where a change occurred, observers detected the change on 67.6 of them. Of these trials, observers went on to correctly identify what had changed on 47.2 of the trials. This meant that approximately 20.4 of the trials were *only-sense*, which was significantly more than the 10.9 trials that would have been predicted by a guessing strategy, *t*(9) = 4.35, *p* = 0.002, *Cohen's d* = 1.37. The data was arcsine transformed before performing the t-test. As before, this result indicates that observers could sometimes detect changes without being able to identify them.

## Experiment 3: Simpler stimuli

The previous two experiments have provided good evidence that for natural images observers can often detect changes without being able to identify what has changed. As discussed in the Introduction, this behaviour would be expected if observers were to monitor the statistics of the scene. Monitoring the scene statistics would help observers identify when a change has occurred but might not provide enough information to allow the observer to identify exactly what has changed. If this reasoning is correct then it should be possible to construct other displays that more readily generate this phenomenon.

A problem with natural images is that by their very nature it is hard to have strict experimental control over them. For example, in a single trial the pose of the subject would be different in the two photographs. While this is realistic in that in a natural settings a subject's pose would be continuously changing, the drawback of this approach is that the photographs would differ not only in the intended change but also along irrelevant dimensions. In the previous experiments, we addressed this potential confound by ensuring that the subject's pose would change by a similar amount in the no change (i.e., catch) trials as in the change trials. Here we wish to address the issue more rigorously by using a simpler stimulus that affords greater control.

In the current experiment, we used an array of disks where each disk was randomly assigned a colour of either red or green with a 50% probability. On change trials three randomly chosen disks all of the same colour, i.e. all red or all green, would change colour, thereby changing the proportion of red and green in the scene. Thus, by monitoring the portion of red and green in the scene we reasoned that it should be easy to detect when a change had occurred but that this information would often not be sufficient to allow the observer to determine which disks had changed colour. This would therefore constitute an existence proof that sensing unidentified changes could be achieved by monitoring the scene statistics.

### Participants

There were ten observers, four male, ages ranged from 18 to 28 years old. As before, all were verified to have normal or corrected-to-normal vision and gave informed consent.

### Stimulus displays and procedure

Each stimulus comprised 30 disks, each disk subtending 1.5 degrees of visual angle. Each disk was assigned the colour red or green with a 50% probability, independent of the colour assignments of the other disks. As before, the initial stimulus was shown to the observer for 1.5 seconds, then there was a 1 second blank interval, followed by a representation of the original array of disks. On change trials, three disks of the same colour would change colour (e.g., three red disks might become green or vice versa). After viewing the second display the observer was first asked to indicate if a change had occurred. If the observer indicated that it had, the second array was presented again and the observer was invited to click on the disk that he thought most likely to have changed colour. If the observer clicked on any one of the three disks that had changed colour then this was counted as a *can-see* trial. Conversely, if the observer correctly identified that a change had occurred but then failed to identify any of the disks that had changed, then this was counted as an *only-sense* trial. Because each disk had been assigned its colour at random the observer could not determine whether or not a change had occurred by simply viewing one of the images and estimating the proportion of red and green. Rather, to detect a change the observer would need to notice when the proportion of red and green changed. In total there were 140 trials of which 40 were catch trials were no change occurred.

### Results

**Figure 5:**
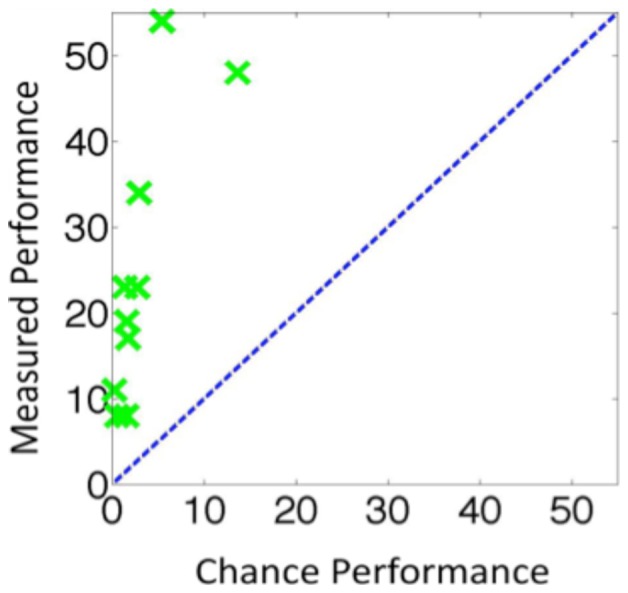
The results for individual observers for Experiment 3 using the same format as Figure 3.

The group results are shown in Figure 2 and the individual results in Figure 5. When a change occurred, observers detected the change on approximately 88.7 trials. Of these trials, observers went on to correctly identify one of the disks that had changed on approximately 64.2 trials. This meant that approximately 24.5 of the trials were *only-sense*, which was significantly more than the 3.2 trials that would have been predicted by a guessing strategy, *t*(9) = 8.99, *p*<0.001, *Cohen d* = 4.27. This data was arcsine transformed before the t-test was performed. This result also held at the individual observer level. For each of the 10 observers, there were more *only-sense* trials than would be expected due to guessing (Figure 3).

## Experiment 4: Changes that do not alter the proportion of red to green in the scene

The previous experiment demonstrated the largest number of *only-sense* trials of all three experiments to date. In Experiment 3 every change trial necessarily caused a large change in the scene statistics, specifically the proportion of red to green in the scene. We expect that observers detect the changes by monitoring the scene statistics [13]. An alternative explanation was that our previous results were due to some other factor. For example, it could be that when observers made a response there was a motor error that would cause them to accidentally select the wrong disk thereby making it look like they did not know which disk had changed even though in reality they did. Alternatively, having indicated that a change had occurred they might then forget what the change was.

Experiment 4 was a control experiment that addressed these concerns. It largely replicated the paradigm of Experiment 3 but utilized changes that would minimise the changes to the scene statistics. Specifically, the changes would not alter the proportion of red to green in the scene. If observers had been monitoring the proportion of red to green to detect unidentified changes then we would expect observers to no longer be able to detect unidentified changes in Experiment 4. Thus, we would not expect any more *only-sense* trials than could be attributed to a guessing strategy. Conversely, had the *only-sense* trials in Experiment 3 been due to motor errors or memory errors, then we would still expect a significant number of *only-sense* trials in Experiment 4.

### Participants

There were ten observers, six male, ages ranged from 18 to 29 years old. As before, all were verified to have normal or corrected-to-normal vision and gave informed consent.

### Stimulus displays and procedure

Observers viewed a display of 15 rectangles, each subtending 1.5 by 3 degrees of visual angle. Half of each rectangle was green and the other half red, allocated at random. As before, the initial stimulus was shown to the observer for 1.5 seconds, then there was a 1 second blank interval, followed by a representation of the original array of rectangles. First the observer was asked if a change occurred and, if the observer indicated that it had, the second display was shown again and the observer was asked to click on the rectangle that the observer was most certain had changed. On change trials, two rectangles changed colour, with the green part becoming red and the red part becoming green. Thus, the overall proportion of red and green was not altered. As before, in total there were 140 trials of which 40 were catch trials were no change occurred.

### Results

**Figure 6:**
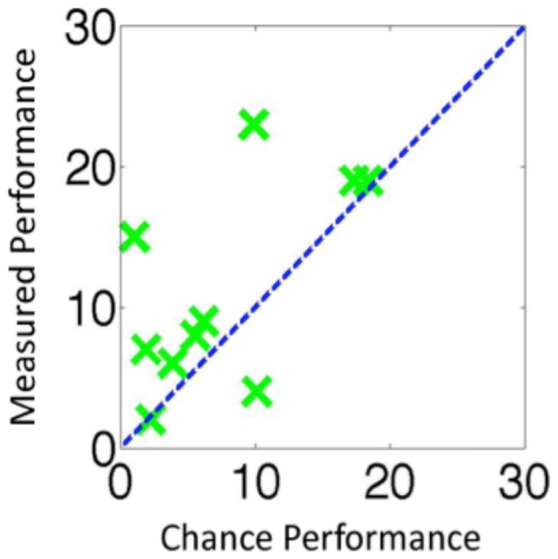
The results for individual observers for Experiment 4 using the same format as Figure 3.

The group results are shown in Figure 2 and the individual results in Figure 6. When a change occurred, observers detected the change on 56.5 trial. Of these trials, observers went on to correctly identify what had changed on 45.3 trials. This meant that in total on 11.2 of trials where a change occurred observers detected but could not identify the change. This was not significantly more than the 7.6 trials that would have been predicted by a guessing strategy, *t*(9) = 1.82, *p* = 0.102. The data was arcsine transformed before performing the t-test. Once the proportion of red to green in the scene was kept constant, we could no longer find any evidence that observers were able to detect changes without also being able to identify them. When a change was noticed, the observers were typically able to identify at least one rectangle that has changed. Furthermore, there were more *only-sense* trials in Experiment 3 than in Experiment 4, *t*(15.2) = 2.49, *p* = 0.025, *Cohen's d*  = 1.14, using arcsine transformed data, equal variances not assumed. This is further evidence that in Experiment 3 observers were detecting changes based on the scene statistics, specifically the proportion of red to green in the scene, and that the large number of *only-sense* trials recorded in that experiment were not due to motor errors or memory errors.

## General discussion

The results of our first two experiments demonstrated that observers were able to detect changes in images of faces even when they could not reliably identify which aspect of the face had changed. By using a one-shot paradigm with catch trials, we were able to rule out the possibility that observers achieved this by using a verification strategy [16] or a guessing strategy [22]. Instead, we argued that it was likely that observers were monitoring the scene statistics and detecting changes in that manner [13]. Experiments three and four provided additional support for this hypothesis using simplified stimuli that we could more rigorously control. In Experiment 3, changes to the scene resulted in changes to the scene statistics, specifically the proportion of red to green in the scene. Observers were readily able to detect that a change had occurred but were less able to identify any one disk that had changed, leading to a large number of *only-sense* trials. We argued that this occurred because detecting that the proportion of red to green in the scene had changed would reliably allow the detection of a change, but would not indicate which disks had changed. Experiment 4 followed up on Experiment 3 by considering changes that would not alter the scene statistics as much. Specifically, these changes would not alter the proportion of red to green in the scene. In Experiment 4, we found that there were no more *only-sense* trials that would be expected to occur due to a guessing strategy. We argued that since monitoring the proportion of red to green in the scene would not help the observer detect changes, the observer would instead have to memorize individual objects. Thus, whenever a change was noticed the observer would necessarily know which object had changed. This would explain why we did not observe any more *only-sense* trials than could be attributed to chance in that experiment. Experiment 4 also ruled out the possibility that the large number of *only-sense* trials recorded in Experiment 3 were due to motor errors or memory errors. Had this been the case we would have expected a significant number of *only-sense* trials in Experiment 4.

These results are broadly consistent with a previous study that employed a very different paradigm, comparative visual search [18]. Galpin et al. similarly found that observers were less likely to sense a change that could not be explicitly identified when pairs of items were swapped, as occurred in Experiment 4, as opposed to when individual items were altered, as occurred in Experiment 3. Furthermore, they found that *sense-only* trials were most likely to occur with uniform arrays, which is consistent with our finding that the most *sense-only* trials occurred in Experiment 3.

Our model of observer behaviour could be described as a high threshold model in that it assumes that a change will never be detected if it does not occur [28]. False alarms are assumed to be due to guessing, not false detections. The model further assumes that if an observer detects a change then they also necessarily know where the change occurs. Because we were able to show that this model did not provide an adequate account of our observers' data we were able to conclude that our observers sometimes had only partial information about the changes that occurred; that is, enough information to determine that a change had occurred but not enough to determine what has changed. Our analysis is therefore consistent with a previous study that shows that high threshold models do not provide an accurate account of change detection [28]. Our analysis goes beyond this previous study by showing that observers can sometimes detected changes without being able to identify or localize them.

It is important to emphasize the limitations of our findings. First, we have not investigated the precision with which an observer can localise an unidentified change. What we have shown is that often an observer can detect that a change has taken place but not be able to identify exactly which object has changed. This should not be taken to mean that the observer has no idea where the changing object is located but rather that he does not know the location precisely enough to be able to identify the changed object. Unfortunately, from none of our experiments is it possible to derive estimates of how precisely an unidentified object can be localised. A second issue is that our experiments do not address the question of the conscious awareness of the change. In particular, our experiments do not prove that when an observer failed to identify a change the observer had no idea at all what the change was. For example, in Experiment 3 the observer would often have some idea of what the change was, e.g. some disks had changed from green to red causing there to be more red in the scene. It was just that this knowledge was insufficient to identify any of the disks that had changed from green to red.

We are not the first authors to claim that observers can sometimes detect changes without being able to identify them. Nakayama and Silverman [14] first demonstrated this phenomenon using sinusoidal gratings. Rensink [15] made a similar claim using natural images however, as discussed in the Introduction, this claim was later disputed [16]. Busch, Frund and Hermann [17] used EEG to investigate the neurophysiological differences that occur when observers can identify what has changed as opposed to merely detecting that a change has occurred. In the later case, the authors found that in particular the N2pc activity was greatly reduced. As N2pc activity is associated with attention, this suggests that detection in the absence of identification may require less attention that when a change is identified. Galpin, Underwood and Chapman [18] found that when changes were *identified*, rather than simply *detected*, observers made more comparative saccades, which is also indicative of the observers paying greater attention to the region that was changed. This suggests that when observers pay increased attention to the region that changes they are more likely to be able to identify what has changed, rather than just detect that a change has occurred.

In this paper we have argued that change detection without identification is probably mediated by sensitivity to scene statistics. However, there could in principle be alternative processes at play. For example, Rensink [15] claimed that some observers possess “mindsight” which is defined as the ability to detect changes before one is able to identify them. Rensink suggested two candidate processes that might mediate mindsight. One possibility is that it may be based on a representation of scene layout. Observers would detect changes by noticing that the layout had changed. The second possibility suggested by Rensink is that seeing (i.e., identifying) a change may involve forming a coherent percept of the object that is changed, a process that would presumably involve attention [29]. Without attention, only the underlying components of the objects, i.e. the features, could be detected which may provide enough information to detect a change, but not enough to allow the observer to identify which object has been changed. This second possibility is very similar to the one that we have been advocating in this paper.

The term “mindsight” was invented in analogy to the phenomenon of blindsight. In blindsight, a patient with damage to the primary visual cortex reports not being able to see stimuli located in the corresponding part of the visual field [30]. However, some of these patients are still able to detect the occurrence of visual transients [31], a phenomenon sometimes referred to as type 2 blindsight [32]. While this type of detection could also be described as mindsight, it is clearly distinct from the form of mindsight studied here as in our experiments an intervening blank screen ensured that none of the changes were signalled by visual transients. Furthermore, in blindsight, patients are better at localizing changes than detecting them whereas in our Experiment 3 the converse occurred.

Our findings also stand in apparent contrast to those that suggest that observers may sometimes be able to detect changes implicitly. It is well known that encoding and retrieval of information can occur independently of conscious awareness in amnesic patients [33], [34]. Similarly, in normal observers a stimulus that does not reach conscious awareness may still influence behaviour (e.g. [35], [36], [37]). Fernandez-Duque and Thornton [23] demonstrated a related phenomenon in the context of change blindness (see also [38], [39]–[41]). In their experiment observers saw an array of rectangles, some of which were horizontal and the rest were vertical. A blank interval was then shown followed by the original array except that on half the trials one of the rectangles changed orientation. The observers were first asked whether they had noticed any change at all. Then two of the rectangles were cued and the observer was asked which had changed. Even on those trials where the observers claimed not to have noticed any change, they were still at above chance levels at indicating which of the two rectangles had changed. The conclusions of this study were subsequently disputed by Mitroff, Simons and Franconeri [42] who essentially argued that even when the observer reported not noticing a change, he might still have some knowledge that may help him guess at the location of the change at above chance level. For example, he might know of some objects in the scene that definitely did not change.

Even if implicit change detection were shown to be a real phenomenon it would still not necessary conflict with our findings. For example it could be that detection and localization/identification are two separate processes. Our experiments demonstrated that observers could sometimes detect changes without being able to localize them. Conversely, implicit change detection would demonstrate that observers can sometimes localize changes even when they cannot detect them. Taken together, these two lines of research would suggest that there is a double disassociation between detection and localization. Of course, this reasoning only holds if implicit change detection is shown to be a real phenomenon [42].

It is generally agreed that if an observer happens to attend to one of the items that is changed then under most circumstances that observer will usually be able to identify that change [1], [5]. For example, when Luck and Vogel [43] had observers monitor three items for a colour change, observers were able to identify colour changes with an accuracy of 97%. Consequently, in those trials in Experiment 3 where the observer was unable to identify any of the three disks that had changed colour, we can be confident that the observer had not attended to any of those disks that had changed. That on 68% of these trials (i.e., those trials where a change occurred but could not be identified by the observer), the observer was still able to detect that a change had occurred shows that change detection can occur even when the items that change are not attended. This constitutes direct evidence that detection can operate in the absence of attention, as has previously been hypothesized [15], [44].

## Conclusions

In this study we have provided direct behavioural evidence that observers can regularly detect when a change has occurred without necessarily being able to identify what has changed. Indeed, when the display was chosen appropriately as in Experiment 3, all our observers demonstrated this ability. Crucially, our data cannot be attributed to the observers' reporting bias [22] or to observers employing either a verification strategy or a guessing strategy [16]. We found that this ability to detect unidentified changes is not unique to images containing faces and it seems to occur primarily when the changes alter the scene statistics. For example, in Experiment 4 where the changes did not alter the scene statistics, there were no more *only-sense* trials than would be expected due to chance. Monitoring the scene statistics may provide observers with enough information to determine when a change has occurred but not enough to precisely localise which object has changed. It is possible that the purpose of detection is to alert the observer to the possible presence of a change so that the observer then knows to search for the change using focal attention.
